# Enfortumab Vedotin‐Related Skin Toxicities: Insights From Clinical and Histopathological Analysis

**DOI:** 10.1111/1346-8138.17901

**Published:** 2025-08-14

**Authors:** Yuki Kuma, Makiko Kido‐Nakahara, Yoko Kuba‐Fuyuno, Takashi Matsumoto, Yoshinao Oda, Masatoshi Eto, Takeshi Nakahara

**Affiliations:** ^1^ Department of Dermatology, Graduate School of Medical Sciences Kyushu University Fukuoka Japan; ^2^ Department of Urology, Graduate School of Medical Sciences Kyushu University Fukuoka Japan; ^3^ Department of Anatomic Pathology, Graduate School of Medical Sciences Kyushu University Fukuoka Japan

**Keywords:** Enfortumab vedotin, immune checkpoint inhibitors, skin toxicity, urothelial carcinomaantibody‐drug conjugate

## Abstract

Enfortumab vedotin (EV), targeting Nectin‐4, is effective in advanced urothelial carcinoma but frequently causes skin toxicities. This study examined the possible contribution of prior immune checkpoint inhibitor (ICI) therapy and higher EV doses to EV‐related skin toxicity (EVST) development. Histopathological findings suggested both direct keratinocyte damage and immune‐mediated mechanisms. These insights may improve clinical management of EVST.

## 1 Introduction

Urothelial carcinoma (UC) has poor survival outcomes in advanced stages. Enfortumab vedotin (EV), an antibody‐drug conjugate (ADC) targeting Nectin‐4, was approved for third‐line treatment since 2021 and for first‐line treatment in combination with pembrolizumab since 2024 in Japan. Despite its efficacy, EV‐related skin toxicities (EVST) frequently complicate treatment. Understanding these dermatologic adverse events is essential for developing effective management strategies, especially as EV becomes increasingly utilized in combination therapies. This study investigates factors and histopathological findings associated with EVST.

## 2 Methods

We retrospectively reviewed 22 patients with metastatic or unresectable UC who were treated with EV monotherapy following ICI in the Department of Urology at our institution between January 2022 and December 2023. EV‐associated skin toxicity (EVST) during treatment and their clinical and histopathological features were further analyzed.

Skin toxicity was assessed based on clinical evaluation. In most cases, dermatologic evaluation was performed by dermatologists and graded according to the Common Terminology Criteria for Adverse Events version 4.03 (CTCAE v4.03). In a few patients who were evaluated solely by urologists, skin toxicity grading was retrospectively determined by the reviewing dermatologist based on medical record documentation. Skin biopsies were obtained from affected lesions and reviewed by board‐certified pathologists and dermatologists. Immunohistochemical staining was performed using antibodies against CD4, CD8, CD68, and CD163. Statistical comparisons of characteristics between groups were conducted using the Wilcoxon rank‐sum test.

### 2.1 Ethical Approval

This study was approved by the Institutional Review Board of Kyushu University Hospital (Approval No. 23361) and conducted in accordance with the Declaration of Helsinki. Written informed consent was obtained from all patients for data collection and publication, including clinical images.

## 3 Results

**FIGURE 1:**
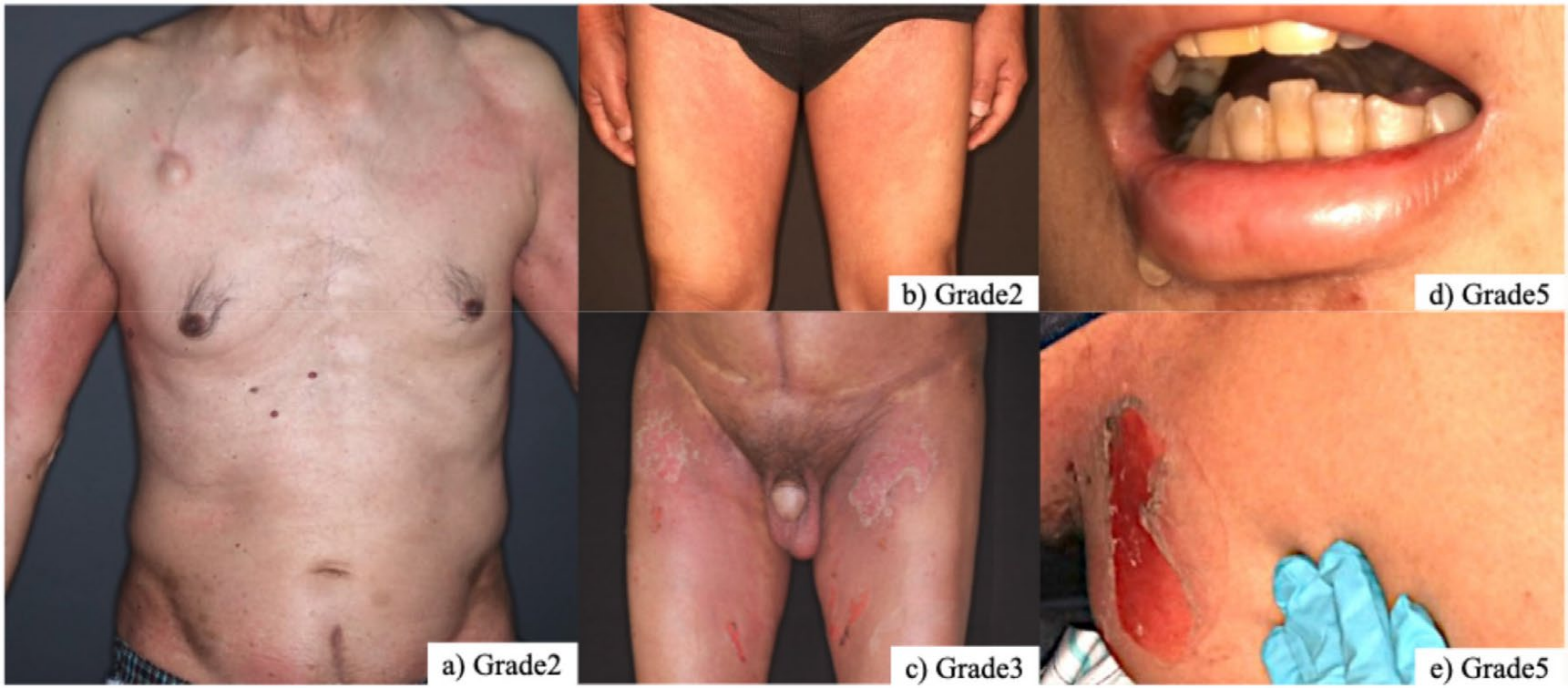
Clinical findings of EV‐induced skin toxicity. (a) Grade 2: Pruritic erythema symmetrically distributed in intertriginous areas. (b) Grade 2: Erythema with severe pruritus and decreased sweating extending from the groin to the thigh. (c) Grade 3: Diffuse erythema with intense pruritus involving the trunk and extremities; partially re‐epithelialized erosions are also observed. (d, e) Grade 5 (TEN‐like clinical picture): Erosions observed on the oral mucosa and on the skin, extending from the left upper back to the axillary region.

All 22 patients had received ICI prior to the administration of EV. Three patients were treated with nivolumab, six with avelumab, and 13 with pembrolizumab. Among the 22 cases reviewed, skin toxicities were observed in 14 patients (63.6%). EVST was significantly associated with higher EV doses and prior ICI cycles (Table 1). Symptoms appeared during the first cycle (median: Day 8). Common findings included erythema localized to intertriginous and flexural areas (92.9%) and pruritus (92.9%). Other dermatologic findings included truncal rash (50.0%), alopecia (50.0%), blisters and erosions (21.4%), and oral erosion (7.14%) (Figure 1). One patient presented with oral erosions as the initial symptom, which progressed to a Stevens‐Johnson syndrome (SJS)/toxic epidermal necrolysis (TEN)‐like course and ultimately resulted in fatality due to multiple organ failure. In this TEN‐like case, after the third administration of EV (Day 15), oral erosions appeared on Day 20, followed by blistering and erosions in the axillae, inguinal region, abdomen, and popliteal fossae (Figure S1). Around the same time, the patient developed grade 4 neutropenia, fever, liver dysfunction, and disseminated intravascular coagulation (DIC). No other known culprit drugs associated with conventional TEN were administered to the patient prior to the onset of the TEN‐like eruption. Systemic management including hydrocortisone sodium succinate 50 mg every 6 h (Days 22–24) was administered in the ICU, but the patient ultimately died of multiorgan failure on Day 24.

**TABLE 1:** Clinical characteristics and presence of skin toxicities.

Clinical parameters	*n* = 22		*p*
Skin toxicity	Present	Absent	
	*n* = 14 (63.6%)	*n* = 8 (36.4%)	
Age (years)			
Mean (years)	70.4	77.9	*p* > 0.05
Median (range)	72.5 (52–88)	76 (70–85)	
EV dose (mg/day)			
Mean	74.7	61.6	*p* < 0.05*
Median (range)	77 (53–90)	65 (46–72)	
ICI administrations			
Mean	10	4	*p* < 0.01**
Median (range)	9 (4–21)	4 (2–7)	

*Note:* Using Wilcoxon's rank sum test.

Abbreviations: EV, enfortumab vedotin; ICI, immune checkpoint inhibitor.

* *p* < 0.05, statistically significant. ** *p* < 0.01, statistically significant.

Patients with EVST were further classified into two groups, Grade 1–2 and Grade 3–5, based on the percentage of body surface area affected by the rash, according to NCI‐CTCAE v4.03. A comparison of clinical parameters between these two groups revealed no significant differences (Table S1).

**FIGURE 2:**
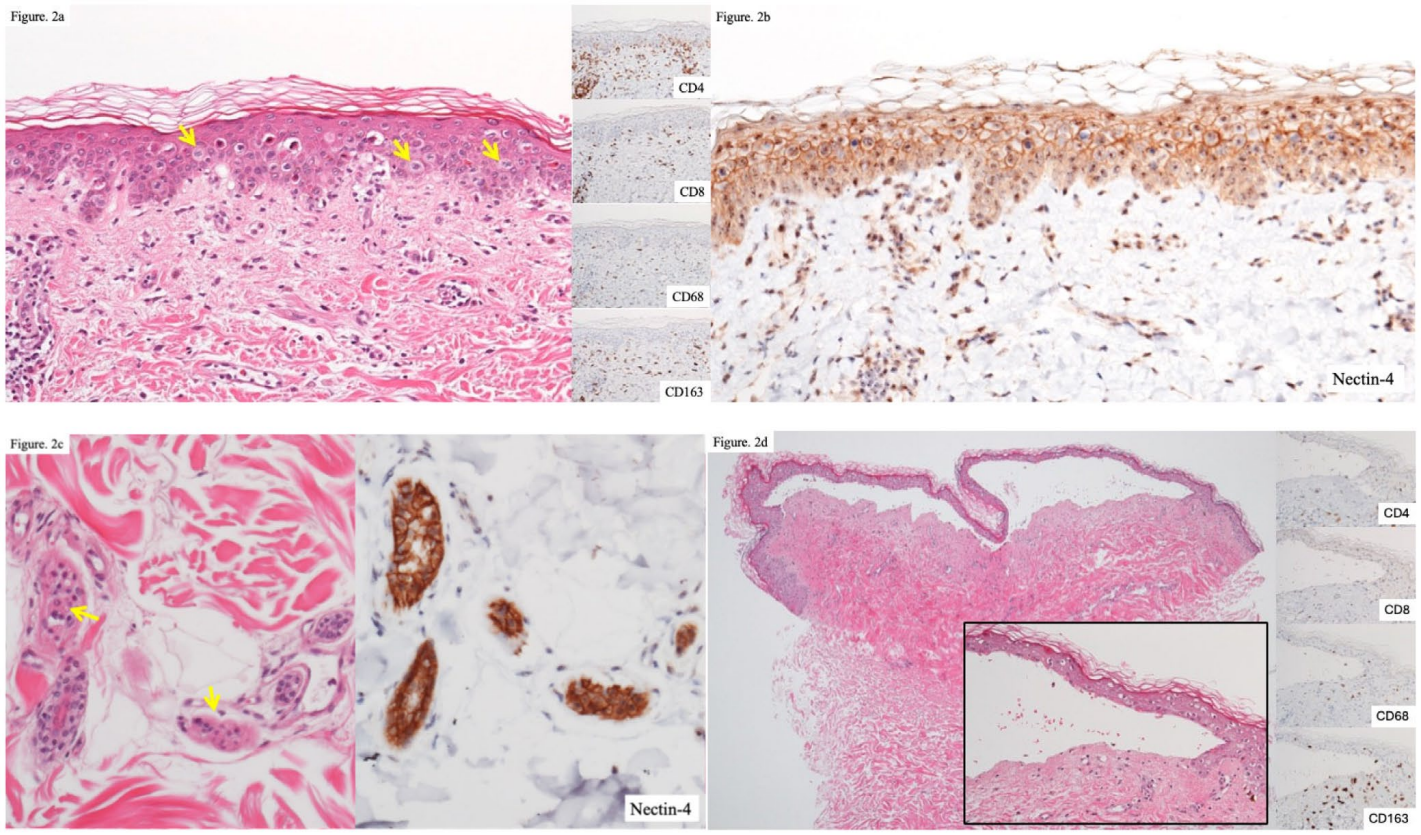
Histopathological findings. (a) In the histopathological findings, extensive apoptosis of keratinocytes, ring‐shaped or stellate mitotic figures (indicated by arrows), and eosinophilic infiltration were observed (×200, HE). (b) In immunohistochemistry, Nectin‐4 was positive on the cell membranes of keratinocytes (×200). (c) Degeneration of sweat glands was observed in the dermis, characterized by eosinophilic cytoplasm and unclear glandular structures (indicated by arrows) (×200, HE). The sweat glands also showed positivity for Nectin‐4 (×200). (d) In the case with a TEN‐like course, subepidermal blisters were observed, with minimal infiltration of inflammatory cells in the blister cavity and the dermis beneath the blister (×40, HE). Immunohistochemical staining revealed the presence of CD163‐positive cells only (×100).

Skin biopsies, including a TEN‐like case (*n* = 10), revealed extensive keratinocyte apoptosis and numerous ring‐ or stellate‐shaped atypical mitotic figures within the epidermis (Figure 2a, Figure S1b). Epidermal edema was observed without evidence of acantholysis. The epidermal‐dermal interface showed liquefactive degeneration, and eosinophilic infiltration was prominent in the perivascular and interstitial areas of the dermis. Immunostaining identified numerous CD4+ cells in both the epidermis and dermis (Figure 2a). In immunohistochemistry, Nectin‐4, the target of EV, was positive on the cell membranes of keratinocytes in all cases (Figure 2b). The severity of skin lesions was categorized into two groups, as presented in Table 1. The corresponding histopathological findings were subsequently evaluated and summarized in Table 2. All nine cases with assessable epidermal pathology exhibited keratinocyte apoptosis and atypical mitosis. Quantification of keratinocyte apoptosis at 200× magnification revealed no significant difference in apoptosis counts between the two severity groups. In four cases within the Grade 1–2 group, degeneration of sweat glands was observed in the dermis, characterized by eosinophilic cytoplasm and unclear glandular structures (Figure 2c). No inflammatory cells were detected around the affected sweat glands. The observed sweat glands also tested positive for Nectin‐4. Seven of 10 cases demonstrated eosinophilic infiltration in the dermis, which was observed in both grade groups. However, the case with a TEN‐like course showed no significant inflammatory infiltration in the blister cavity or the underlying dermis (Figure 2d). Another case of grade 3 skin toxicity involved erosions on the thighs and required hospitalization in our department. Pathological examination revealed epidermal loss. Due to concern for progression to a severe clinical course and the lack of established treatment guidelines for such cases, steroid pulse therapy with methylprednisolone 1000 mg/day for 3 days was administered. The patient responded promptly to the treatment, and skin symptoms improved rapidly. In the remaining cases, skin symptoms improved with oral prednisolone (0.3–0.5 mg/kg/day), antihistamines, and topical application of very strong or strongest class corticosteroids.

**TABLE 2:** Histopathological findings and severity of skin lesions.

Histopathological features in epidermis (*n* = 9)^a^ and dermis (*n* = 10)	Total	Grade 1–2	Grade 3–5
		Skin involvement < 30%	Skin involvement ≥ 30% or SJS/TEN‐like
Epidermis (keratinocytes)	*n* = 9	*n* = 5	*n* = 4
Rounded or star‐shaped nuclear fragments	9	5	4
Apoptosis	9	5	4
+: 1–10 cells per 200× field	1	0	1
++: 10–20 cells per 200× field	2	2	0
+++: > 20 cells per 200× field	6	3	3
Interface changes at the dermo epidermal junction	9	5	4
Dermis	*n* = 10	*n* = 5	*n* = 5
Eosinophilic infiltration			
None	3	1	2
Mild to moderate	5	4	1
Severe	2	0	2
Sweat gland changes	4	4	0

Abbreviation: SJS/TEN, stevens‐johnson syndrome/toxic epidermal necrolysis.

^a^ Grade 3 with erosion case was excluded from histological analysis due to lack of epidermis, resulting in *n* = 9.

## 4 Discussion

UC is a malignancy arising from the transitional epithelium lining the renal pelvis, ureters, bladder, and urethra. It is associated with poor survival outcomes, with a 5‐year survival rate for stage IV UC below 20%. While systemic chemotherapy has been the standard treatment for nearly 30 years, the introduction of ICIs in 2017 and the approval of EV in 2021 have significantly advanced therapeutic options in Japan [1].

EV is an ADC targeting Nectin‐4 and delivering monomethyl auristatin E (MMAE), a microtubule‐disrupting agent [2]. Its high tumor selectivity and relatively low systemic toxicity have established EV as a next‐generation anticancer agent. Nectin‐4 is overexpressed in several carcinomas, including UC, but is also expressed in normal tissues, such as the epidermis, skin appendages, esophageal mucosa, and gastric mucosa. While EV shows potential as a novel therapy for skin appendage tumors [3], its association with cutaneous toxicity has been widely reported. Most EVST occurs during the first treatment cycle and commonly affects intertriginous and flexural areas, extremities, and the trunk [4]. In the phase III EV‐301 trial, EVST was observed in 139 of 296 patients (47.0%), with Grade 3 or higher skin toxicity in 43 cases (14.5%) [5]. A post hoc analysis identified a higher incidence of severe skin toxicity in Japanese patients compared to non‐Japanese patients [6], prompting the Pharmaceuticals and Medical Devices Agency in Japan to recommend treatment interruption for Grade 2 toxicities until they resolve to Grade 1 or lower.

In our study, all EVST cases occurred during the first treatment cycle, consistent with previous reports. Notably, half of the EVST cases were Grade 3 or higher, and the majority required dermatologic intervention. We observed that patients who developed EVST had received a greater number of prior ICI administrations and, in some cases, higher doses of EV. Although the number of cases is limited, this raises the possibility that immune modulation by ICIs may contribute to EVST occurrence, possibly through sustained immune activation or altered keratinocyte responses.

Histopathological evaluation revealed that all EVST cases exhibited abnormal mitotic figures reminiscent of findings seen in taxane‐based chemotherapies and prior EV reports [7, 8]. It has also been reported that EV can induce keratinocyte apoptosis and starburst mitoses even in clinically intact skin [9]. CD4+ T cells, CD68+ macrophages, and eosinophils were predominant in both the epidermis and dermis, whereas CD8+ T‐cell infiltration was relatively sparse. These findings support a mechanism in which keratinocyte apoptosis is initiated by MMAE‐induced cytotoxicity, with secondary immune cell recruitment contributing to tissue damage. The mechanism underlying eosinophilic infiltration in EV‐related skin toxicity is not fully elucidated. However, it may be attributable to antigen presentation initiated by the extracellular release of MMAE, leading to downstream immune activation.

While most cases are manageable with grade‐based intervention strategies, a critical concern is the relatively high incidence of life‐threatening severe skin reactions, such as SJS and TEN, compared to other agents [10]. Although several reports from Japan and abroad have described TEN or TEN‐like eruptions in association with EV administration [11, 12, 13, 14], many of these lack histopathological confirmation or sufficient diagnostic information. Therefore, current evidence remains inconclusive, and a cautious, neutral interpretation is warranted when evaluating such cases.

One notable case presented with a TEN‐like clinical picture. In this patient, we observed a marked absence of inflammatory cells in the blister cavity, along with dermal infiltration by CD163+ M2 macrophages—cells known to play roles in both inflammation and tissue repair. This infiltration pattern is consistent with previous reports, including that by Sasaki et al. [15]. Conversely, classical TEN may also exhibit limited inflammatory cell infiltration, particularly in early‐stage lesions, making histopathological differentiation between classical TEN and EV‐associated TEN‐like reactions inherently challenging. Therefore, diagnosis should be guided by clinical presentation, temporal association with EV exposure, and treatment response, rather than relying solely on histopathologic findings.

According to the latest Japanese supplementary guidelines for the management of SJS/TEN, EV‐induced severe cutaneous reactions may present with TEN‐like clinical features but are thought to involve a distinct pathogenic mechanism from true TEN. The guidelines caution against diagnosing SJS/TEN based solely on epidermal detachment and advise against routine systemic immunosuppression in cases attributable to cytotoxic antineoplastic agents. Instead, supportive therapies, including topical corticosteroids and oral antihistamines, are recommended. We propose that future studies should investigate the temporal dynamics of serum cytokine profiles in patients receiving EV, as well as evaluate specific inflammatory markers in skin biopsy samples to better understand and potentially mitigate EV‐associated skin toxicities. Further studies are warranted to optimize treatment strategies tailored to the unique mechanisms of EV‐associated skin toxicity.

## 5 Conclusion

EV‐induced skin toxicities were linked to higher doses and prior ICI therapy, involving both direct keratinocyte damage and immune‐mediated mechanisms. Pembrolizumab with EV was recently approved in Japan for first‐line treatment of advanced UC. Dermatologists will play a critical role in managing these reactions, particularly as EV use expands in combination therapies.

## Conflicts of Interest

The authors declare no conflicts of interest.

## Supporting information


**Figure S1:** Clinical and histopathological features of the case with a TEN‐like course that resulted in fatality. (a) Erythema and flaccid blisters observed on the intertriginous areas and feet. (b) Histopathological examination revealed extensive keratinocyte apoptosis, ring‐shaped or stellate mitotic figures (indicated by arrows), similar to the findings in Figure 2a (×200, HE).

**Table S1:** Clinical characteristics and severity of skin toxicities.

## Data Availability

The data that support the findings of this study are available on request from the corresponding author. The data are not publicly available due to privacy or ethical restrictions.
