# Supplementary material for: Enfortumab Vedotin‐Related Skin Toxicities: Insights From Clinical and Histopathological Analysis
Source: J Dermatol. 2025 Aug 14;52(10):1584–8. doi: 10.1111/1346-8138.17901 (PMC12530469; doi:10.1111/1346-8138.17901)
Supplement: Supplementary file 1 — Figure S1: Clinical and histopathological features of the case with a TEN‐like course that resulted in fatality. (a) Erythema and flaccid blisters observed on the intertriginous areas and feet. (b) Histopathological examination revealed extensive keratinocyte apoptosis, ring‐shaped or stellate mitotic figures (indicated by arrows), similar to the findings in Figure 2a (×200, HE). [file JDE-52-1584-s001.docx]

**SUPPLEMENTARY FIGURE. 1**
