# Supplementary material for: Enfortumab Vedotin‐Related Skin Toxicities: Insights From Clinical and Histopathological Analysis
Source: J Dermatol. 2025 Aug 14;52(10):1584–8. doi: 10.1111/1346-8138.17901 (PMC12530469; doi:10.1111/1346-8138.17901)
Supplement: Supplementary file 2 — Table S1: Clinical characteristics and severity of skin toxicities. [file JDE-52-1584-s002.docx]

**Supplementary Table 1:** Clinical Characteristics and Severity of Skin Toxicities

| **Clinical parameters** | **n=14** | |  |
| --- | --- | --- | --- |
| **skin toxicity** | **Grade 1-2**  **Skin involvement <30%** | **Grade 3-5**  **Skin involvement ≥30% or SJS/TEN-like** | **P-value** |
|  | n=7 (50%) | n=7 (50%) |  |
| Age (years) |  |  |  |
| Mean (years)  Median (range) | 71.4  72 (54-88) | 69.7  73 (52–79) | P > 0.05 |
| EV dose (mg/day） |  |  |  |
| Mean  Median (range) | 71.9  75 (53-90) | 77.3  77 (67-86) | P > 0.05 |
| ICI administrations |  |  |  |
| Mean  Median (range) | 11  10 (6-17) | 9  7 (4-21) | P > 0.05 |

Using Wilcoxon's rank sum test
